# Investigation of the genetic diversity of domestic *Capra hircus* breeds reared within an early goat domestication area in Iran

**DOI:** 10.1186/1297-9686-46-27

**Published:** 2014-04-17

**Authors:** Seyed Mohammad Farhad Vahidi, Ali Reza Tarang, Arif-un-Nisa Naqvi, Mohsen Falahati Anbaran, Paul Boettcher, Stephane Joost, Licia Colli, Jose Fernando Garcia, Paolo Ajmone-Marsan

**Affiliations:** 1 Agricultural Biotechnology Research Institute of Iran (ABRII), North branch, Rasht 41635-4115, Iran; 2 Department of Biological Sciences, Karakoram International University, Gilgit, Pakistan; 3 School of biology and Center of Excellence in Phylogeny of Living Organisms, University of Tehran, PO Box 14155-6455, Tehran, Iran; 4 Department of Biology, Norwegian University of Science and Technology, N-7491 Trondheim, Norway; 5 Animal Production and Health Division, Food and Agriculture Organization of the United Nations, Rome 00153, Italy; 6 Laboratory of Geographic Information Systems, School of Civil and Environmental Engineering (ENAC), Ecole Polytechnique Fédérale de Lausanne (EPFL), 1015 Lausanne, Switzerland; 7 Istituto di Zootecnica and Biodiversity and Ancient DNA - BioDNA - Research Centre, Università Cattolica del Sacro Cuore, Piacenza, Italy; 8 Departamento de Apoio, Produçãoe Saúde Animal, Laboratório de Bioquímica e Biologia Molecular Animal, Rua Clóvis Pestana, Universidade Estadual Paulista, Aracatuba, Brazil; 9 Animal Production and Health Section, International Atomic Energy Agency, A-1400 Vienna, Austria

## Abstract

**Background:**

Iran is an area of particular interest for investigating goat diversity. Archaeological remains indicate early goat domestication (about 10 000 years ago) in the Iranian Zagros Mountains as well as in the high Euphrates valley and southeastern Anatolia. In addition, mitochondrial DNA data of domestic goats and wild ancestors (*C. aegagrus*or bezoar) suggest a pre-domestication management of wild populations in southern Zagros and central Iranian Plateau. In this study genetic diversity was assessed in seven Iranian native goat breeds, namely Markhoz, Najdi, Taleshi, Khalkhali, Naini, native Abadeh and Turki-Ghashghaei. A total of 317 animals were characterized using 14 microsatellite loci. Two Pakistani goat populations, Pahari and Teddy, were genotyped for comparison.

**Results:**

Iranian goats possess a remarkable genetic diversity (average expected heterozygosity of 0.671 across loci, 10.7 alleles per locus) mainly accounted for by the within-breed component (*G*_ST_ = 5.9%). Positive and highly significant *F*_IS_ values in the Naini, Turki-Ghashghaei, Abadeh and Markhoz breeds indicate some level of inbreeding in these populations. Multivariate analyses cluster Iranian goats into northern, central and western groups, with the western breeds relatively distinct from the others. Pakistani breeds show some relationship with Iranian populations, even if their position is not consistent across analyses. Gene flow was higher within regions (west, north, central) compared to between regions but particularly low between the western and the other two regions, probably due to the isolating topography of the Zagros mountain range. The Turki-Ghashghaei, Najdi and Abadeh breeds are reared in geographic areas where mtDNA provided evidence of early domestication. These breeds are highly variable, located on basal short branches in the neighbor-joining tree, close to the origin of the principal component analysis plot and, although highly admixed, they are quite distinct from those reared on the western side of the Zagros mountain range.

**Conclusions:**

These observations call for further investigation of the nuclear DNA diversity of these breeds within a much wider geographic context to confirm or re-discuss the current hypothesis (based on maternal lineage data) of an almost exclusive contribution of the eastern Anatolian bezoar to the domestic goat gene pool.

## Background

Goats are multi-purpose animals that produce milk, meat and fiber and also serve other beneficial roles. In particular, they contribute to the economy of farmers living in arid and semi-arid regions, including southern Iran [1]. Although goat products are often cheaper than sheep products in the market place, goats are favored in the most marginal areas of Iran, where they are easier to manage and better adapted to harsh climate and ecological conditions than sheep. According to the latest livestock census, conducted in 2008, the Iranian caprine population is around 25 300 000 animals (http://faostat.fao.org). Iranian goats are mainly reared in traditional systems by small holders. Since nomadic tribes are almost completely economically dependent on animal rearing, these stakeholders play an important role in the conservation of animal genetic resources, especially of small ruminants.

Genetic diversity is an essential component for population survival, evolution, genetic improvement and adaptation to changing environmental conditions [2]. Information on genetic diversity is therefore necessary to optimize both conservation and strategies for the use of animal genetic resources, to meet future market demands and improved production systems. Molecular tools permit the characterization of genetic resources at the DNA level. Because of favorable characteristics, such as abundant number, high polymorphism and co-dominant inheritance, microsatellite DNA markers have been extensively used for a number of applications in livestock genetics, including parentage testing, breed classification, conservation genetics and also to assess genetic variation and structure within and among populations [3].

This study was undertaken to examine the pattern of microsatellite variation within and among seven Iranian goat breeds. The resulting information may be used in national plans for sustainable improvement and conservation of goat genetic resources. This research was carried out as part of the IAEA-FAO joint program “Characterization of genetic resources in small ruminants in Asia” (D3.10.25), which aimed at developing methodologies, generating information and formulating decision support systems to analyze phenotypic and molecular genetic diversity, develop microsatellite and related technologies, and enable the development and implementation of national and regional strategies for optimum use and conservation of small ruminants in Asia (http://www-naweb.iaea.org/nafa/about-nafa/index.html).

## Methods

### DNA sampling

**Figure 1:**
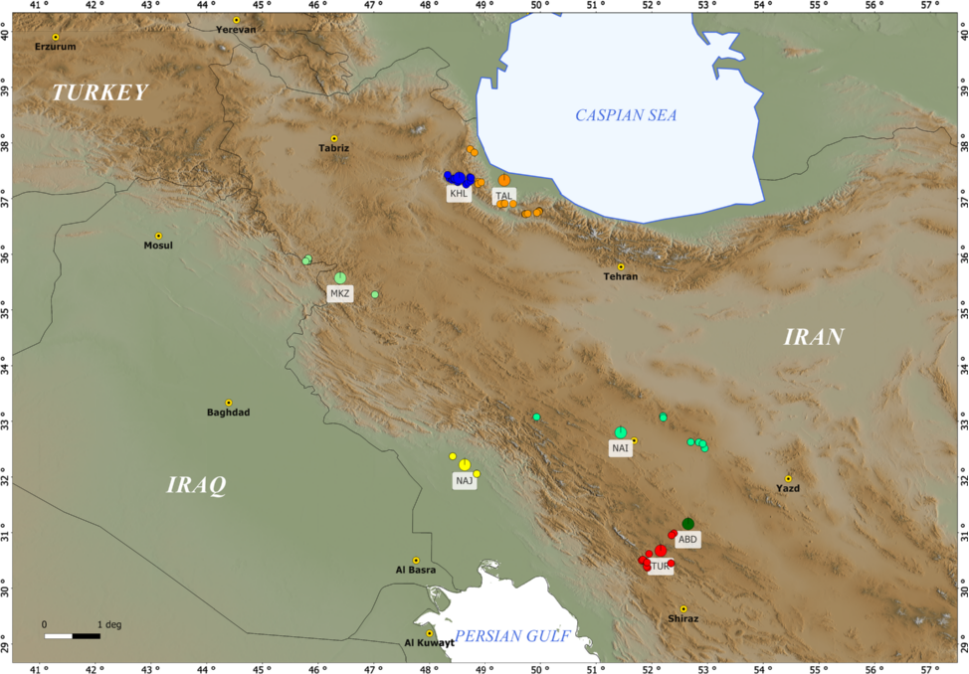
Distribution of Iranian goat populations surveyed in this study.

**Figure 2:**
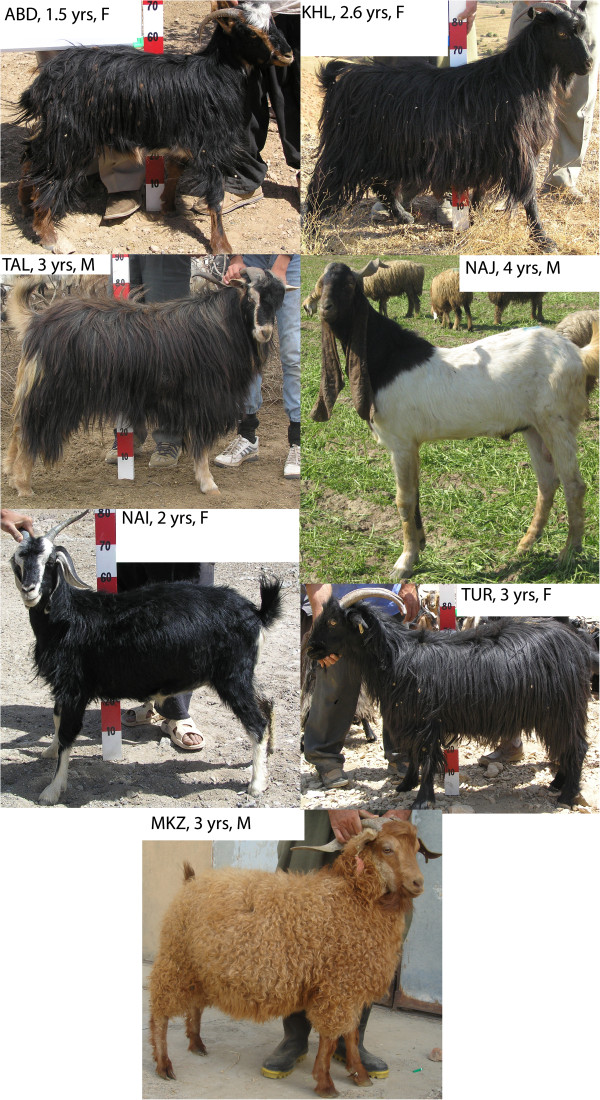
**Morphologies of animals from the different Iranian goat breeds analyzed.** Breed ID, age of animal in years (yrs) and age are shown in the white boxes.

Iranian goats were sampled in six different areas that extended from the north of Iran, in the Alborz Mountains, south of the Caspian Sea, to the far western border of Iran, in the northern Zagros Mountains, to southern Zagros, along the mountain range. Seven indigenous goat breeds were mainly distributed in six provinces: Gilan, Ardabil, Isfahan, Fars, Kurdistan and Khuzestan (Table 1). A maximum number of five samples per flock were collected from an average of 11 flocks per breed (min = 5, max = 18). Two Pakistani goat breeds, collected from the Punjab province, were also included in the dataset for comparison. The geographic distribution of Iranian breeds sampled in this study is depicted in Figure 1 and their typical phenotype in Figure 2.

**Table 1:** Sampling information and basic parameters of genetic diversity for nine goat breeds (13 microsatellite markers)

						**Allelic diversity**					**Genetic diversity**		
**Country**	**Province**	**Population name**	**Code**	**N**	**NF**	**TNA**	**NEA (SD)**	**MNA (SD)**	**A**_**r**_	**NPA (Freq. range)**	***H***_**E **_**(SD)**	***H***_**O **_**(SD)**	***F***_***IS***_
Iran	Gilan	Taleshi	TAL	34	17	98	3.75 (1.34)	7.54 (3.26)	5.88	8 (0.015-0.045)	0.710 (0.030)	0.710 (0.021)	0.001
	Ardebil	Khalkhali	KHL	41	18	95	4.00 (1.68)	7.31 (3.12)	5.78	1 (0.012)	0.713 (0.035)	0.696 (0.020)	0.024
	Isfahan	Naini	NAI	39	10	97	3.61 (1.69)	7.46 (2.30)	5.64	4 (0.013-0.053)	0.670 (0.043)	0.622 (0.022)	0.072**
	Fars	Turki-Ghashghaei	TUR	38	11	104	3.58 (1.53)	8.00 (2.24)	5.99	3 (0.013-0.015)	0.681 (0.036)	0.644 (0.021)	0.055*
	Fars	Abadeh	ABD	30	8	87	3.93 (1.47)	6.69 (2.10)	5.83	1 (0.019)	0.720 (0.035)	0.602 (0.026)	0.166***
	Kurdestan	Markhoz	MKZ	38	9	83	3.52 (1.50)	6.38 (2.50)	5.18	4 (0.013-0.129)	0.658 (0.050)	0.615 (0.022)	0.067**
	Khuzestan	Najdi	NAJ	20	5	61	2.65 (1.06)	4.69 (1.65)	4.25	0	0.586 (0.046)	0.611 (0.031)	-0.045
Pakistan	Punjab	Teddy	TED	38	-	77	3.39 (1.16)	5.92 (1.44)	4.91	4 (0.026-0.132)	0.678 (0.036)	0.655 (0.021)	0.035
	Punjab	Pahari	PAH	39	-	79	3.12 (1.31)	6.08 (2.29)	4.79	0	0.625 (0.047)	0.612 (0.021)	0.021
	Mean						3.50 (1.41)	6.67 (2.32)	6.17		0.671 (0.040)	0.641 (0.023)	

N = sample size; NF = number of sampled flocks; TNA = total number of alleles; NEA = mean number of effective alleles; SD = standard deviation; MNA, mean number of alleles; *A*_r_ = allelic richness based on a minimum sample size of 12 diploid individuals; NPA = number of private alleles; *H*_E_ = expected heterozygosity; *H*_O_ = observed heterozygosity; *F*_IS_ = population inbreeding coefficient; significant values are as indicated: *P < 0.05, **P < 0.01, ***P < 0.001.

### Microsatellite DNA analysis

The salting-out method [4] was used to isolate genomic DNA from blood samples of 317 animals from the seven Iranian and two Pakistani goat breeds. Fourteen microsatellite markers were chosen from the list recommended by the FAO [5]. Forward primers were end-labeled with fluorescent dyes (6-FAM, VIC, NED and PET) [see Additional file 1]. Polymerase Chain Reaction (PCR) was carried out on 50-100 ng of genomic DNA in a 15 μL reaction containing 1.5 μL of 10× PCR buffer, 1 μL of 20 mM dNTPs, 0.2 μL of each primer at a concentration of 10 μM and 1 unit DNA polymerase. Each marker was amplified individually. The “Touchdown” PCR protocol used an initial 5 min denaturation step at 95°C, followed by 3 cycles at 95°C during 45 s and 60°C during 1 min, 3 cycles at 95°C during 45 s and 57°C during 1 min, 3 cycles at 95°C during 45 s and 54°C during 1 min, 3 cycles at 95°C during 45 s and 51°C during 1 min and 20 cycles at 92°C during 45 s and 48°C during 1 min, a 45 s extension step at 72°C and a final 10 min extension step at 72°C. Microsatellite genotypes were visualized with the ABI PRISM 3130XL DNA Analyzer (Applied Biosystems, USA) and alleles were scored using GeneMapper™ software Version 3.7 (Applied Biosystems, USA). In total, 11 samples from four populations (KHL, TAL, MKZ and NAI) were genotyped in duplicate, to evaluate data quality and repeatability. To ensure correct genotype scoring, visual inspection was carried out independently by two experienced operators and all conflicting scores sorted out.

### Statistical analyses

Unbiased estimates of genetic diversity (*H*_E_), observed heterozygosity (*H*_O_) and mean number of alleles (MNA) were calculated using the Microsatellite Toolkit [6]. The probability-test (exact HW test), used to assess deviations from Hardy-Weinberg equilibrium (HWE) for each locus and population and for loci over all populations, was performed with Genepop 4.0 using a Markov chain of 100 000 steps and 1000 dememorization steps, 500 batches and 10 000 iterations per batch [7]. *P* values from multiple comparisons were corrected using a Bonferroni correction [8]. Null alleles can decrease estimates of genetic diversity and inflate genetic differentiation [9]. To estimate the potential frequency of null alleles (r) for each locus in each breed, we used the EM algorithm of [10] in the software FreeNA [11]. This method assumes that deviations from HWE do not result from other causes (e.g. from the Wahlund effect). Values of r ≤ 0.2 are expected not to cause significant problems in the analyses [9]. FSTAT program version 2.9.3 [12] was used to estimate Wright’s fixation indices [13]. Standard errors were generated using the jack-knife method over loci and populations. The same software was used to calculate the inbreeding coefficient (*F*_IS_) for each population and a pairwise *F*_ST_ distance matrix. The rarefaction technique of El Mousadik and Petit (1996) [14] was used in FSTAT to calculate allelic richness (number of alleles in a sample of standardized size). Cervus 3.0 was used to calculate the polymorphism information content (PIC) of each locus [15]. The number of effective alleles (Ne) per locus in each population [16] was calculated with the POPGENE 1.32 software [17]. The average number of effective migrants exchanged per generation (gene flow, Nm) was calculated with the following formula: Nm = (1 - *F*_ST_)/(4*F*_ST_) as applied in Genetix 4.05 [18]. This software was also used to estimate unbiased Nei’s coefficient of gene variation (*G*_ST_) [19]. Beaumont and Nichols’s approach [20], implemented in LOSITAN [21] was used to detect loci under selection. This software uses computer simulation to detect loci for which the genetic diversity within (heterozygosity) and between populations (F_ST_) do not conform to the prediction of a simple island model obtained by coalescent simulations [22]. Similarity in *F*_ST_/*H*_E_ values for all loci indicates a shared demographic history. Loci showing unusually large amounts of differentiation may mark regions of the genome that have been subject to directional selection, while loci showing unusually small amounts of differentiation may mark regions of the genome that have been subject to balancing selection [23]. All loci outside a 99.5% confidence interval were removed and the mean *F*_ST_ was calculated again. A final run included all loci. The infinite allele model and 95 000 simulations were used in this calculation. Hierarchical analysis of molecular variance (AMOVA) was conducted with geographical location as grouping factor, using Arlequin 3.11. Goat breeds were spatially partitioned into five groups including (A) TAL and KHL, (B) NAI, TUR and ABD, (C)MKZ, (D)NAJ and (E) TED and PAH (Pakistani populations). AMOVA was used to measure the extent of hierarchical genetic differentiation among the locations, among populations within a location, and among individual within a population [24].

Three approaches were used to analyze the genetic relationships among individuals and populations: (i) genetic distances and dendrograms, (ii) model-based cluster analysis, and (iii) principal components analysis (PCA). A dendrogram was constructed using the Neighbor-Joining (NJ) algorithm [25] in DISPAN [26], based on Nei’s genetic distance (*D*_A_) [27]. Trees were edited with MEGA4 [28]. The Bayesian model-based clustering method implemented in STRUCTURE software [29] was used to investigate population structure and define clusters of individuals on the basis of multi-locus genotypes. The number of assumed clusters (*K*) varied between 2 and 11. For each *K*, 10 independent runs were performed with a burn-in of 10^5^ and Markov chain Monte Carlo length of 2 × 10^5^ iterations under an admixture model with correlated allele frequencies and no prior information on the population of origin (popinfo = 0). The assignment probabilities were compiled for multiple runs in the program CLUMPP, which addresses multimodality and/or label-switching in run comparisons [30]. We used the Greedy algorithm to increase computational speed, set the pairwise similarity matrix to G’ and ran 1000 random repeats of the data for the determined value of *K*. The results of STRUCTURE analyses were depicted using the software Distruct [31]. The estimate of the best *K* was calculated as described by Evanno et al. [32] using Structure Harvester v.0.6.92 [33]. PCA was performed using XLSTAT software (Addinsoft, Paris) to summarize and visualize the structure of data described by several quantitative variables, while obtaining the uncorrelated factors between them.

## Results

### Genetic diversity

**Figure 3:**
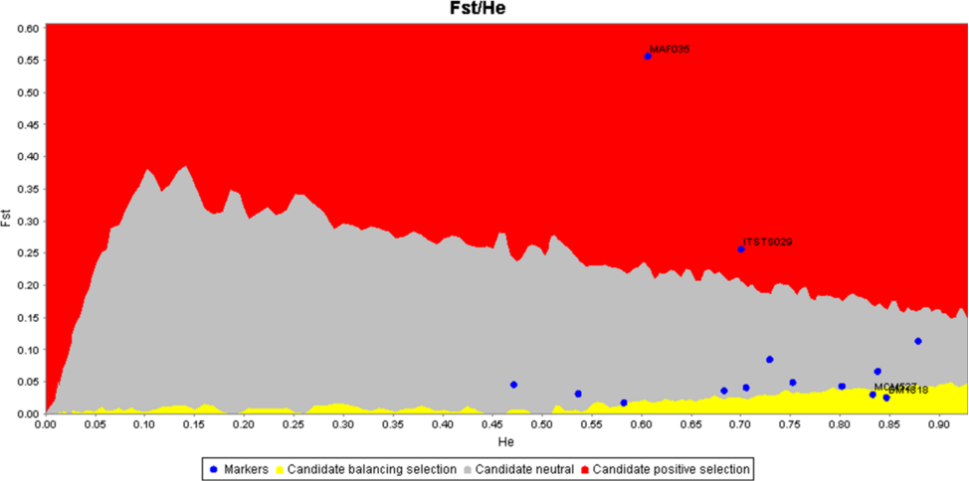
**Graphical output from LOSITAN.** Outliers are tagged with labels.

A total of 154 genotypes were produced on the 11 animals, which were genotyped twice. All genotypes were identical between replicates indicating a very high repeatability of the genotyping and scoring procedures adopted. A total of 150 alleles were detected at the 14 microsatellite loci in the nine goat breeds. Allele number ranged from five (MAF035) to 18 (ILSTS029) per locus and the average number was equal to 10.7. Most loci displayed a high degree of polymorphism, as revealed by the PIC values that ranged between 0.435 (INRA0132) and 0.851 (MAF70), with a mean of 0.67. Relevant information per locus, such as the range of allele sizes, location on chromosomes, sequence and label of the primers, number of alleles (observed and effective), PIC and deviation from HWE, are presented in Additional file 1 [see Additional file 1]. Seven out of 14 loci deviated from HWE after sequential Bonferroni correction (*P* < 0.05). Five populations, NAI, TUR, ABD, MKZ and TED, showed deviation from HWE for at least one locus (*P* < 0.05). Nine of the 126 locus × population combinations revealed significant departures from HWE. Several estimates of the frequency of null alleles (r) were greater than 0.11, i.e. for BMS1494, ILSTS029 and MAF035 [see Additional file 2]. As previously reported, values of r ≤ 0.2 are expected not to cause significant problems in the analysis [9]. The only exception was the ILSTS029 locus in the ABD population, for which r = 0.23. Frequency distributions of two loci (BM1818 and MCM527) were indicative of balancing selection at the 99.5% probability level, whereas directional selection was suggested at the loci MAF035 and ILSTS029 (Figure 3). Since there was a strong suggestion that the MAF035 locus was under selection because of its low heterozygosity and low number of alleles (Table 1; Figure 3), it was excluded from further analyses. Conversely, to avoid too much loss of information, all other markers were retained, including BM1818 that was reported to flank QTL (Quantitative Trait Loci) for fertility and milk traits in cattle [34]. Based on the raw data for 13 markers, the largest mean number of alleles (MNA) was observed in TUR (8.00) and the smallest in NAJ (4.69). NAJ had the fewest samples (N = 20), but this trend remained consistent for corrected allelic richness (*A*_r_), which was also greatest in TUR (5.99) but smallest in NAJ (4.25). The mean of the effective number of alleles per locus and population ranged from 2.65 (NAJ) to 4.00 (KHL) (Table 1). The mean effective number of alleles had a global mean of 3.50 across all loci, which was remarkably lower than the mean observed number of alleles (10.7). The average unbiased expected heterozygosity over all loci ranged from 0.58 (NAJ) to 0.72 (ABD). The overall mean of gene diversity was equal to 0.671 (Table 1). ABD (0.602) and TAL (0.710) had the lowest and the highest observed heterozygosity, respectively (Table 1). The mean *F*_IS_ across all loci and breeds was equal to 0.052. *F*_IS_ were significantly greater than zero in NAI, TUR, ABD (*P* < 0.001) and MKZ (*P* < 0.01), which indicated inbreeding in these breeds (Table 1).

### Genetic differentiation

The global *F*_ST_ obtained by the jack-knife method over loci was equal to 0.062 ± 0.016 and significantly different from zero (*P* < 0.001). Wright’s *F*-statistics were calculated for each of the 13 microsatellite loci across the nine breeds [see Additional file 3]. The AMOVA revealed that most of the molecular variance occurred within breeds (92.90%) while it represented 3.73% among geographic groups and 2.07% among breeds within a geographic group (Table 2). Although small, the group/breed components were both statistically significant (*P* < 0.01 and *P* < 0.001, respectively). Very similar results were obtained by clustering breeds according to the groups identified in the STRUCTURE analyses.

**Table 2:** AMOVA of the goat breeds based on 13 microsatellite loci

**Structure**	**Source of variation**	**Degrees of freedom**	**Sum of squares**	**Squared value**	**Percentage of variation**
7 Iranian breeds	Among groups	4	118.043	0.16213	3.73**
	Among populations within groups	4	42.895	0.08975	2.07***
	Among individuals within populations	308	1277.477	0.05648	1.30^NS^
	Within individuals	317	1279.000	4.03470	92.90***
	Total	633	2717.415	4.34305	

NS = not significant; **P < 0.01; ***P < 0.001.

### Genetic relationship and population structure analysis

**Figure 4:**
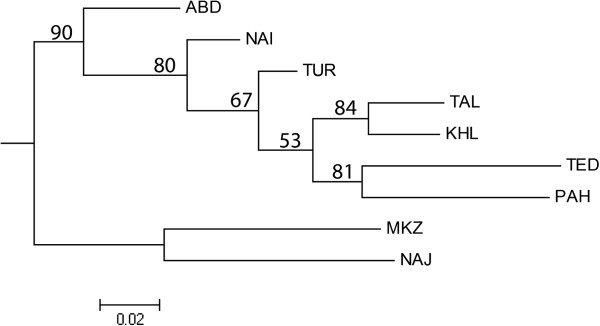
**Neighbor-Joining tree based on *D***_**A **_**genetic distances for nine populations.** Numbers at the nodes are bootstrap values based on 1000 permutations.

**Figure 5:**
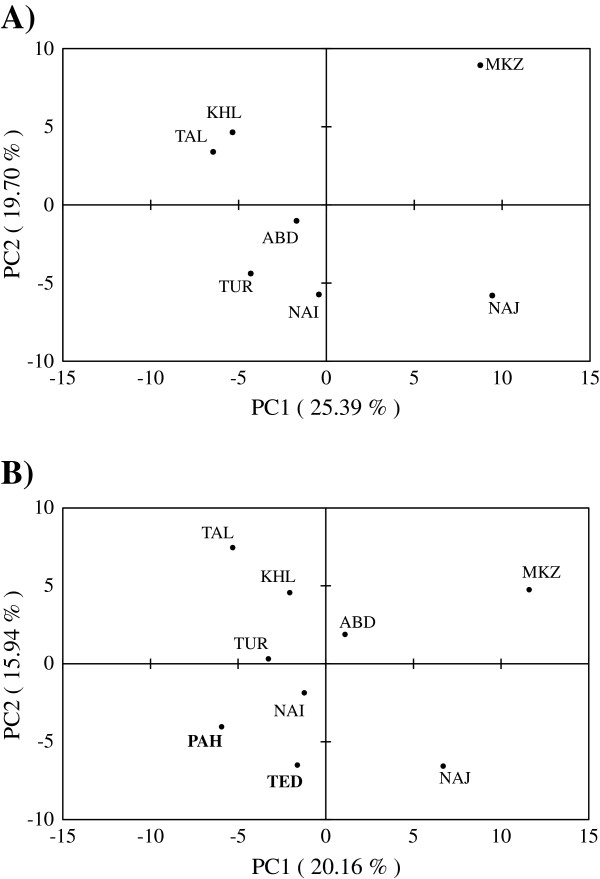
**Principal component analysis.** The principal components were extracted by correlation coefficients of Pearson, based on allele frequencies. **A)** PCA analysis of seven Iranian breeds. **B)** PCA analysis of nine goat breeds (Iran and Pakistan).

**Figure 6:**
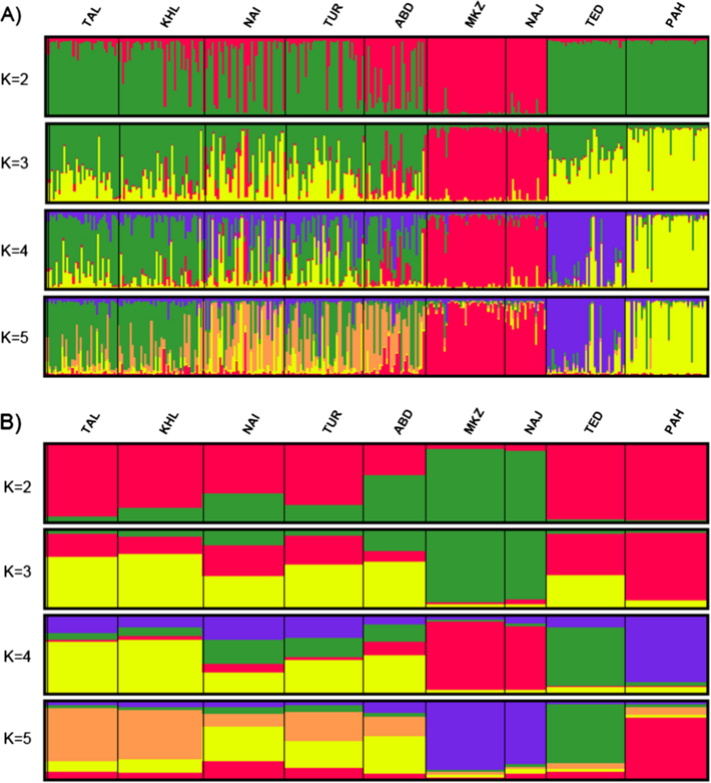
**Clustering assignments of the nine goat breeds obtained by STRUCTURE analyses.** Each of the 317 animals is represented by a thin vertical line that is divided into segments the size and color of which correspond to the relative proportion of the animal genome assigned to a particular cluster; breeds are separated by thin black lines. **A)** Estimated population structure displayed with individual *Q*-scores. **B)** Estimated population structure displayed with population average *Q*-scores.

The *F*_ST_ values between breed pairs ranged from 0.0041 for the TAL-KHL pair to 0.1622 between PAH and MKZ breeds. Pairwise estimates of *F*_ST_ for north (TAL and KHL) and center (NAI, TUR and ABD) of Iran were not significant. For all remaining breed pairs, *F*_ST_ values were highly significant (*P* ≤ 0.001) (Table 3).The number of migrants per generation (Nm) ranged from 60.23 between TAL and KHL populations to 1.29 between MKZ and PAH populations (Table 3). A gene flow of 3.62 was obtained between the two Pakistani goat breeds, PAH and TED. Nei’s genetic distances (*D*_A_) ranged from 0.0521 between TAL and KHL to 0.2760 between NAJ and PAH. The two Pakistani breeds (TED and PAH) showed the lowest *D*_A_ distance with TUR. Nei’s standard genetic distance (*D*_S_) ranged from 0.0108 (TAL-KHL) to 0.4269 (PAH-MKZ). TED and PAH showed the lowest *D*_S_ distances with the Iranian KHL and TUR goat breeds [see Additional file 4]. A NJ tree was constructed based on *D*_A_ genetic distances (Figure 4). Most of the bootstrap values were high (> 70%), which indicated that the dendrogram was very robust. According to the NJ tree, Iranian populations showed a clear clustering, in agreement with the traditional breed classification and geographical origin. An exception was the inclusion in the same cluster of MKZ and NAJ breeds in spite of their rather distant distribution areas, adaptation to different climates and different production purposes. However, they are separated by long branches, indicating that, although they share a common ancestry, they still remain well differentiated. In the *D*_A_ tree, the two Pakistani breeds (TED and PAH) cluster with the northern Iranian breeds (TAL and KHL), even if the branch lengths indicate that they remain rather distant from the Iranian pool and from each other. PCA grouped Iranian breeds in accordance with the NJ trees (Figure 5A).The first component separates populations according to a northwest to southeast gradient, while the second has no clear geographic component. Northern (TAL and KHL) and central (TUR, NAI and ABD) breeds form two groups, while, the western (MKZ) and southwestern (NAJ) breeds clearly separate from each other and also from the other breeds. Inclusion of the two Pakistani breeds (Figure 5B) does not change the configuration of the Iranian breeds. However, in this case, PAH and TED appear to be closer to the central breeds (NAI and TUR) rather than to the northern ones (TAL and KHL) as in the NJ representations. In the STRUCTURE analysis, *K* = 5 resulted as the most appropriate number of partitions [See Additional file 5 and Additional file 6]. Analysis at *K* = 5 divided Iranian goats into three clusters formed by populations from the north (TAL and KHL), center (NAI, TUR and ABD) and west (MKZ and NAJ) of Iran. The two Pakistani breeds were assigned to two other distinct clusters (Figure 6). Admixture was particularly evident between central and northern Iranian clusters, with some components also contributed by the Pakistani PAH breed, whereas the western Iranian cluster formed a quite distinct gene pool.

**Table 3:** **Pairwise estimates of ***F*_**ST **_**and Nm between nine goat breeds using 13 microsatellite markers**

	**TAL**	**KHL**	**NAI**	**TUR**	**ABD**	**MKZ**	**NAJ**	**TED**	**PAH**
TAL		0.0041^NS^	0.0269	0.0216	0.0297	0.0943	0.0946	0.0455	0.0599
KHL	60.23		0.0260	0.0166	0.0162	0.0810	0.0959	0.0335	0.0609
NAI	9.03	9.35		0.0133^NS^	0.0162^NS^	0.1026	0.0859	0.0488	0.0448
TUR	11.33	14.48	18.54		0.0202^NS^	0.1113	0.1002	0.0379	0.0362
ABD	8.16	15.22	15.18	12.14		0.0879	0.0891	0.0431	0.0574
MKZ	2.40	2.83	2.19	2.00	2.59		0.0846	0.1230	0.1622
NAJ	2.39	2.36	2.66	2.25	2.56	2.71		0.1286	0.1549
TED	5.25	7.20	4.87	6.35	5.55	1.78	1.69		0.0646
PAH	3.92	3.86	5.32	6.65	4.11	1.29	1.36	3.62	

*F*_ST_ estimates above the diagonal are all significant at *P* < 0.001 except those marked NS (not significant); numbers of effective migrants per generation (Nm) are below the diagonal.

## Discussion

Iran is close to, and in the case of goats, within, the main south west Asian livestock domestication center. In fact, archaeological remains indicate an early goat domestication (about 10 000 years ago) in the Iranian Zagros Mountains [35], as well as in the high Euphrates valley and southeastern Anatolia [36]. In addition, analysis of mitochondrial DNA of domestic goats and their wild ancestors (*C. aegagrus*or bezoar) revealed signals of population expansions in wild populations in southern Zagros (Fars Province) and in the central Iranian Plateau (Yazd and Kerman Provinces), likely indicating a pre-domestication management of wild populations [37]. These regions were therefore hypothesized to be the site of origin of one of the mtDNA haplogroups (the C haplogroup) and of “an incipient goat domestication phase”. However, haplogroup C has a modest frequency within the mitochondrial gene pool of modern goats, thus suggesting that these regions may not have contributed much to the molecular variability of domestic goat maternal lines, and definitely much less than eastern Anatolian sites. Therefore, it is highly interesting to investigate the genetic diversity of Iranian farm animals for several reasons: (1) social and economic role that these livestock play within the country, (2) their geographic area of origin and (3) new information on the domestication processes that may arise from the analysis of the nuclear genome of local genetic resources. Particularly intriguing would be to test if nuclear DNA data agree with mtDNA information in estimating a marginal involvement of Iranian and a major contribution of Anatolian gene pools into goat domestication processes.

The analysis of 13 microsatellites resulted in a mean genetic diversity of 0.671 (Table 1). Although comparison with investigations using different marker sets is only indicative, the value we observed is greater than those reported in Swiss goat breeds (0.51 to 0.58) genotyped at 20 microsatellite loci [38] and in 11 indigenous south east Asian goats analyzed with 25 microsatellites (0.43-0.60) [39], but it is slightly lower than those reported in Chinese goat breeds (0.77-0.82) analyzed with six microsatellite loci [40]. However, Di et al. [41] assessed the genetic diversity of nine Chinese cashmere goats, two Iranian goats and one breed from Guinea Bissau using 14 microsatellite markers and found the greatest diversity among the Iranian breeds.

In the present study, expected heterozygosity and allelic richness had the highest values for ABD and TUR (two Iranian goats) with means of 0.72 and 5.99, respectively. We can thus conclude that Iranian goats possess a remarkably high genetic diversity, as expected, for native populations in the vicinity of a domestication center. With the exception of NAJ, the surveyed populations had higher expected than observed heterozygosities. This resulted in positive *F*_IS_ values that were highly significant for NAI, TUR, ABD and MKZ (Table 1), indicating some level of inbreeding in these populations. *F*_IS_ reached the remarkable value of 16.6% in ABD. The presence of null alleles probably contributed to the very high *F*_IS_ value observed in this breed. Conversely the Wahlund effect, did not contribute to increase *F*_IS_ values, since no population substructure was detected in the four inbred breeds by Bayesian cluster analysis. The investigated goat breeds showed a remarkable difference between the effective and the observed number of alleles (sometimes a decrease of more than 50%) [see Additional file 7], due to a very low frequency of many alleles across loci. This effect may result from the combined effects of the exchange of migrants between populations and of the post-domestication population expansion that can still be detected in traditionally managed populations nearby domestication sites, as opposed to western breeds that have likely lost many rare alleles by genetic drift during the process of breed formation.

An overall mean of *G*_ST_ = 5.9% (based on 13 markers) indicated that within-breed diversity accounts for a large part of the total genetic diversity of the breeds investigated. This observation is confirmed by AMOVA and by the low average pairwise *F*_ST_ value (0.062; Table 2 and Additional file 3). This value is similar to that i.e. 0.069 reported by Canon et al*.*[42] but lower than values reported for south-east Asian (0.14; [39]) and Swiss goat breeds (0.17; [38]). The test for neutrality (Figure 3 and Additional file 8) suggested that some loci are under directional (MAF035 and ILSTS029) and balancing selection (BM1818 and MCM527). Since a panel comprising only a few microsatellite markers is of limited interest to identify selection signatures, here the test was used merely to decide if certain markers were to be eliminated from population genetic analyses to avoid biased results [43]. However, these loci might merit further investigation, since they are potentially associated to traits of interest, e.g. the MAF035 locus has been associated with a QTL for carcass traits (percent lean in carcass and total fat) in sheep [44] and BM1818 to QTL for milk and fertility traits in cattle [34].

The distribution of the Iranian breeds described by PCA (Figure 5A) is consistent with the geographical locations of the farms where samples were collected (Figure 1), confirming at the country level that differentiation of diversity in nuclear genomes of goat breeds contains a significant portion of geographic structure, as has already been reported at the continental level [42]. Interestingly, this geographic structure is maintained also in a system of traditional pastoralism, as that present in Iran, in spite of the fact that nomadism and gene flow exist among the populations. The exchange of migrants among populations is, in fact, relevant (Table 3), in particular among the two northern breeds and, to a lesser extent, among the three central breeds. Gene flow is higher within regions compared to between regions and is particularly low between the west and the other regions, due to the topography of the Zagros mountain range. The results of the STRUCTURE analysis are consistent with this interpretation (Figure 6). Western breeds (MKZ and NAJ) form a defined cluster at *K* ranging from 2 to the most probable value (*K* = 5). At this value of *K*, Iranian breeds from the north (TAL and KHL), center (NAI, TUR and ABD) and west and south-west (MKZ and NAJ) form three clusters. The level of admixture is high in breeds from central Iran. It is lower in northern breeds that appear to contain almost identical proportions of ancestral genomes, confirming their high similarity as indicated by genetic parameters in the NJ tree and PCA. The two Pakistani populations constitute two distinct separate gene pools, although they originate from the same area (Punjab province) in Pakistan. Gene flow between these two breeds (Nm = 3.62) confirms STRUCTURE, NJ and PCA analyses and indicates that PAH and TED are distinct, even if some animals from TED seem to have a large portion of their genome in common with PAH. Overall, NAJ and MKZ, although they share a common ancestry at *K* = 5 (Figure 6), seem to be quite distinct from each other, as indicated by their *D*_A_ and *D*_S_ distances [see Additional file 4], low level of gene flow (Nm = 2.71), long branch length in NJ trees (Figure 4) and clear separation in the PCA plot (Figure 5). In fact, common ancestry does not necessarily imply similarity in gene frequencies. Genetics, geographic distance, agro-climatic conditions, phenotype and main use clearly distinguish these two breeds from each other.

MKZ is a breed of the Kurdish areas (Kurdistan province) of Iran (Figure 1). It is a Mohair-producing breed valued for its shiny fine fiber. It is well adapted to withstand the severe winters that occur in western Zagros, with average daily temperatures below 0°C and heavy snowfalls. A recent report indicates that this breed is presently endangered, due to reduction of population size and number of breeding herds. The population size of MKZ was estimated at over 22 000 animals in 1996, but has progressively decreased to around 5000 heads in 2005 [45]. NAJ, a dairy and fleece goat breed from the Arab region (Khuzestan province; Figure 1) is adapted to extremely high temperatures. Morphologically, MKZ and NAJ are clearly different.

The level of population differentiation and genetic structure observed in Iranian goat breeds are clearly different from that observed in Iranian sheep populations (*F*_ST_ = 0.02, unpublished data). This may be due to the massive amount of gene flow occurring in sheep by translocation of superior breeds over a large geographical distance, because of the higher economic importance of sheep compared to goats. Overall, the degree of differentiation at the few microsatellite marker loci used in this study might appear inadequate to represent the degree of differentiation among breeds that is perceived based on physical appearance and other phenotypic traits. However, “neutral” markers such as microsatellites are designed to reconstruct the evolutionary and demographic history of populations and are theoretically “blind” to the effect of natural and anthropogenic selection that is sometimes very effective and rapid in changing morphological and production traits [46]. It has been reported that degree of differentiation in quantitative traits (*Q*_ST_) typically exceeds that observed in neutral marker genes (*F*_ST_) [47], suggesting a prominent role for natural selection in accounting for patterns of quantitative trait differentiation among contemporary populations.

Taken together, all the approaches used to analyze genetic relationship among individuals and populations in this study suggested a high molecular diversity in Iranian goats, with varying levels of genetic distinctiveness among breeds and considerable gene flow between breeds from the same geographic region. Between-breed diversity has a strong geographic component. Iranian goat breeds fall into three clusters, northern, central and western, with the western and southwestern breeds relatively distinct from others. Pakistani breeds show some relationships with Iranian populations, even if their position is not consistent across analyses. Pakistan and Iran are neighbors, connected by the Baluchistan region that is shared by the two countries. There is a long history of contact and mutual influence between the two countries. Agribusiness and livestock exchange have been ongoing for ages, so it is not surprising to find some similarity in the genetic background of Iranian and Pakistani goat breeds.

In conclusion, to maintain the present genetic diversity and structure of these breeds, proper strategies of marker-assisted management need to be designed and implemented. Although a decreasing number of MKZ individuals has been noted, none of these breeds seem to be endangered according to the FAO risk classification system [48]. Therefore, breed management plans should emphasize sustainable use and development, rather than conservation *per se*. One suggested first step is to organize breeders into formal or informal associations, to facilitate development and implementation of genetic resource management strategies. Inbreeding seems to affect some breeds and an organization of breeders may allow for wider exchange of males within breeds, which would address this problem. Conversely, if the breeders express an interest in maintaining genetic purity, gene flow among breeds and regions should be monitored and avoided. Development of more complex strategies would benefit from the analysis of native breeds with high-density marker panels that can distinguish between neutral and selected genomic regions. This additional information would contribute to the decision making process, in particular by identifying patterns of diversity along genomes of neutral (present day) and selected (very near future) genomic regions. Three out of the seven investigated breeds are reared in geographic areas in which mtDNA provided evidence of early domestication. TUR and ABD (southern Zagros, Fars province) and NAI (central Zagros, Isfahan province) fall exactly in the area in which the C haplogroup is observed at high frequency [37]. Interestingly, these breeds are highly variable (Table 1), are placed on basal short branches in the NJ tree (Figure 4), close to the origin of the PCA plot (Figure 5) and, although highly admixed, quite distinct from those reared on the western side of the Zagros mountain range. These observations reveal the necessity for further investigation of goat nuclear DNA diversity within a much wider geographic context, including Turkey, Europe and Asia. Such an investigation would help to clarify the events that occurred in central Zagros and to the west of the Zagros mountain range during domestication, either confirming or re-discussing the current hypothesis based on maternal lineage data of an almost exclusive contribution of the eastern Anatolian bezoar to the domestic goat gene pool.

## Competing interests

The authors declare that they have no competing interests.

## Authors’ contributions

All authors contributed in designing the experiment, developing methods and drafting the manuscript. All authors read and approved the final manuscript.

## Supplementary Material

Additional file 1 **Characteristics of 14 microsatellite markers used to study nine Iranian and Pakistani goat breeds.** The data provided represent the characteristics of the microsatellite markers used to study seven Iranian and two Pakistani goat breeds. N_a_ indicates the number of alleles at each locus, N_e_ is the number of effective alleles, PIC is the Polymorphic Information Content calculated by Cervus 3.0.3 software, *H*_E_ the genetic diversity per locus per population, *P < 0.05, *P < 0.01, ***P < 0.001, NS=not significant. Click here for file

Additional file 2 **Estimated null allele frequencies using the EM algorithm (r).** The data provided represent the estimation of null allele frequencies using the Expectation Maximization (EM) algorithm for each locus and breed. r ≥ 0.2 is bolded. Click here for file

Additional file 3 **Wright’s *F*-statistics for each of the 13 microsatellite loci across nine breeds.** The file contains the estimated *F*-statistics per locus and overall values, across nine goat breeds. ***P < 0.001, the values in parentheses are the standard errors. Click here for file

Additional file 4 ***D***_**S **_**and *D***_**A **_**genetic distances between nine goat breeds based on 13 microsatellite markers.** The data provided represent the *D*_*S*_ and *D*_*A*_ genetic distances between the breeds analyzed, based on 13 loci. Standard genetic distances (*D*_S_) (below the diagonal) and Nei’s genetic distances (*D*_A_) (above the diagonal). Click here for file

Additional file 5 **The calculated measurements using Evanno method to find the best *K *** **based on Structure output.** The Evanno table output was based on 13 microsatellite loci used in the evaluation of the nine goat breeds. Click here for file

Additional file 6 **Representation of the number of ideal clusters identified by Structure software**. The delta K method (Evanno et al. [32]) was examined to find the most likely K. Click here for file

Additional file 7 **The actual and effective number of alleles of 14 microsatellite loci in nine goat breeds.** The file contains the calculated actual and effective number of alleles per locus for each breed. N = actual number of alleles; Ne = effective number of alleles. Click here for file

Additional file 8 ***H***_**E**_**, *F***_**ST **_**and confidence intervals obtained by LOSITAN.** The file contains the fixation index and expected heterozygosity estimated for each locus to identify loci under selection using LOSITAN software. Click here for file
