# Supplementary figures and images for: Investigation of the genetic diversity of domestic Capra hircus breeds reared within an early goat domestication area in Iran
Source: Genet Sel Evol. 2014 Apr 17;46(1):27. doi: 10.1186/1297-9686-46-27 (PMC4044659; doi:10.1186/1297-9686-46-27)

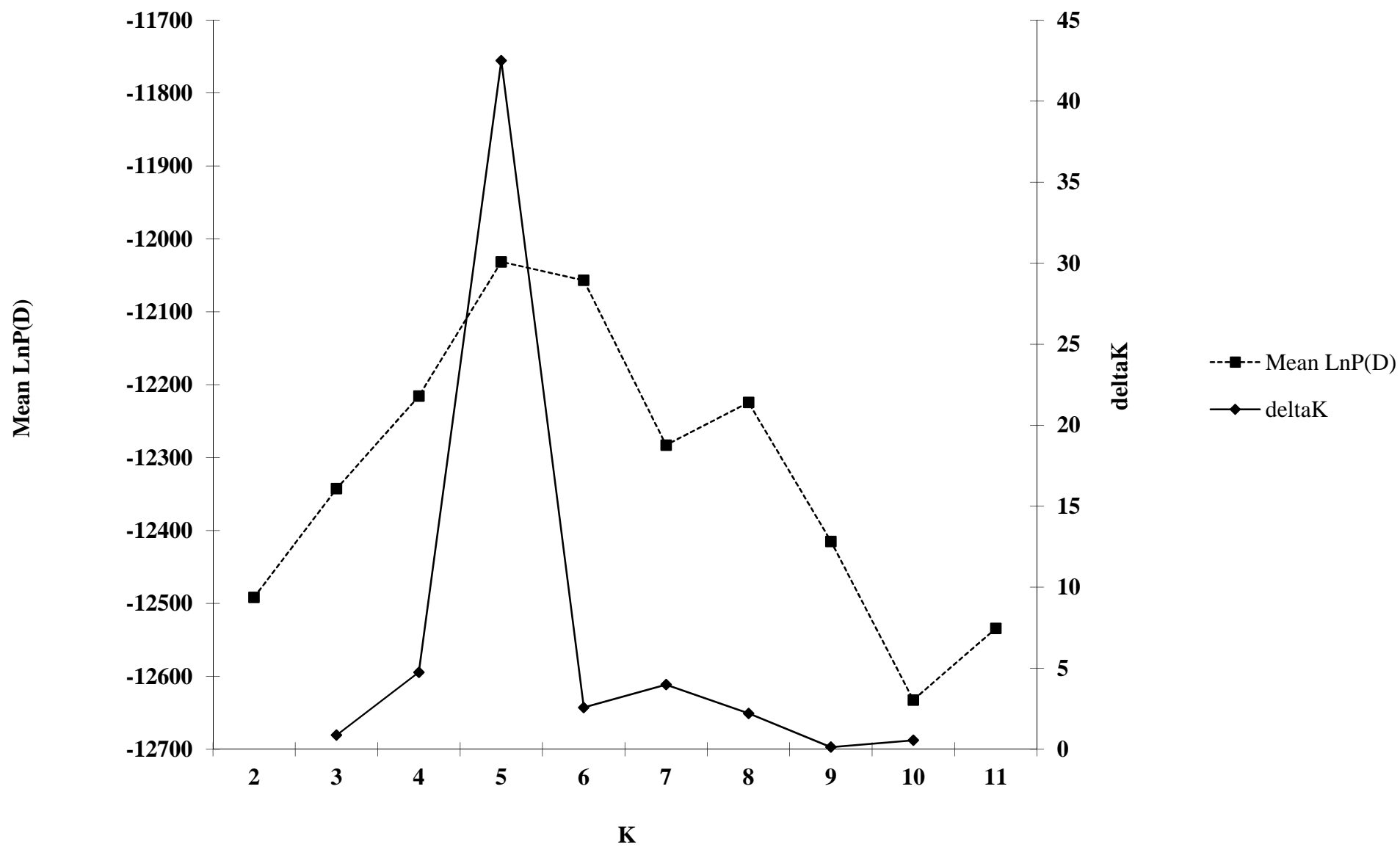

Supplement: Additional file 6 — Representation of the number of ideal clusters identified by Structure software. The delta K method (Evanno et al. [32]) was examined to find the most likely K. [file 1297-9686-46-27-S6.pdf]
